# The New Challenge of Sports Nutrition: Accepting Insect Food as Dietary Supplements in Professional Athletes

**DOI:** 10.3390/foods10051117

**Published:** 2021-05-18

**Authors:** Umberto Placentino, Giovanni Sogari, Rosaria Viscecchia, Biagia De Devitiis, Lucia Monacis

**Affiliations:** 1 ITAF Sports Centre, Office for the Coordination and Management of Professional Athletes, Vigna di Valle, 00062 Roma, Italy; uplacentino@gmail.com; 2 Department of Food and Drug, University of Parma, 43124 Parma, Italy; giovanni.sogari@unipr.it; 3 Department of Agriculture, Food, Natural Resources and Engineering, University of Foggia, 71122 Foggia, Italy; rosaria.viscecchia@unifg.it; 4 Department of Humanities, Literature, Cultural Heritage, Education Sciences, University of Foggia, 71122 Foggia, Italy; lucia.monacis@unifg.it

**Keywords:** food neophobia, disgust, protein source, sport endorsement

## Abstract

Background: The dietary supplements market is growing, and their use is increasing among professional athletes. Recently, several new protein supplements have been placed in the marketplace, including energy bars enriched with insect flour. Edible insects, which are rich in protein content, have been promoted as the food of the future and athletes could be a reference sample for their continued emphasis on higher protein demand. The present study investigated the potential motivations to accept an energy protein bar with cricket flour, among a group of selected Italian professional athletes. A second aim was also to measure how an information treatment about the benefits of edible insects would have impact on acceptance. Methods: 61 Italian professional athletes (27 females) completed a structured questionnaire regarding supplements and eating habits, food neophobia, nutrition knowledge, willingness to taste edible insects and the associated factors. A question about sports endorsement was also posed at the end of the survey. Results: all subjects consumed supplements, generally recommended by medical personnel, even though their general knowledge of nutrition was poor (47.8%). Our main results shown that on a seven-point Likert scale, the protein content (5.74 ± 1.01) and the curiosity about texture (5.24 ± 0.98) were the main drivers to taste the cricket energy bar; whereas the feeling of disgust (5.58 ± 1.08) justified the rejection of tasting insects. In addition, the level of food neophobia increases with age (*p* < 0.05) and reduces willingness to endorse the cricket bar (*p* < 0.05). Male athletes (4.47 ± 1.69) were more likely to endorse the product than females (3.3 ± 1.49). An increase in willingness to taste was observed after the information treatment (z = 4.16, *p* < 0.001). Even though the population under investigation is unique, it is important to mention that this study involves a relatively small and convenience sample, and therefore generalizability of the results should be done with caution.

## 1. Introduction

Food plays a key role in acquiring the best physical condition and in ensuring optimal athletic performance. Physical activity, sports performance, and recovery after exercise are favored by optimal nutrition and professional athletes should be able to fully cover their nutritional needs through the consumption of foods, in adequate quantity and quality [1]. However, it is common among professional athletes to look for dietary supplements to ensure an optimal performance. Frequent competitive commitments and high intensity daily workouts could justify the use of supplements in professional athletes, especially in the case of inadequate diets or situations of temporary inability to maintain correct eating habits [2].

The global sports nutrition market accounted for several billion dollars and is expected to grow significantly because of its increasing demand from athletes and sportspersons in the near future [3]. The size of sport nutrition market in Europe is expected to achieve USD 15.12 billion by 2025. It refers to the consumption of sports drinks, bars, powders and other food supplements to improve physical performance [4].

However, a high proportion of these marketed supplements lack available evidence of their efficacy [5] and these products are often used without a full understanding of the potential benefits, negative side effects, and risks associated. As reported by the World Anti-Doping Agency (WADA), between 10 and 25% of currently marketed supplements reportedly contain prohibited substances, contributing to 6–9% of the total doping offenses [6,7]. Previous literature has shown gaps in knowledge about effective nutrition and supplementation among coaches and athletes [1]. It goes without saying that greater nutritional knowledge may improve dietary practices and food choices [8], reducing the risk of using prohibited substances.

The prevalence rate of the intake of sports supplements is generally high and similar among different countries. However, several demographic variables affect the proportion of athletes that consumed dietary supplements. Gender, age, level of competition, type of sport and professionalism influenced this proportion. It exceeds 60% and increases with professionalism [2,9,10].

The main reasons for consuming supplements are to enhance performance, speed, strength, and power, or to simply improve health. In a recent study, most of the athletes reported that supplements are safe and can be consumed without any risk. Male athletes were more likely to obtain information about the use of supplements mainly from a coach or physician [11].

Professional athletes are often used as brand ambassadors in marketing campaigns due to their high popularity and power to promote consumer engagement. They can use their highly visibility to endorse healthy and environmentally friendly foods, including supplements. Recently on the market of food supplements, there has been focus drawn to products derived from “novel foods”, including insects. In particular there has been a growing prevalence of energy bars enriched with insect protein, in particular cricket flour [12,13].

### Factors Influencing Edible Insects’ Acceptance

In the last decades, there has been a growing interest among the scientific community, private industry, the general public, and the media about edible insects as human food [14,15]. Especially, after the publication of the report on edible insects as food and feed in 2013 by the Food and Agriculture Organization of the United Nations [16], and the new Regulation of the European Union 2015/2283 on novel foods [17], there has been an increasing number of scientific publications investigating the European consumer acceptance towards entomophagy [18,19], especially in Italy [20]. In addition, more recently the European Food Safety Authority (EFSA) published a scientific opinion on the safety of the first edible insect, i.e., *Tenebrio molitor larva* [21]. This will likely favor the development of the edible insect market also in the EU. Globally, a recent study forecasts that this market will reach almost USD 8 billion, with a volume of 730,000 tons by 2030 [22]. Thus, it seems plausible to predict a potential increase in the consumption, especially with regard to products containing invisible insects as ingredients such as in energy protein bars [23]. In particular, a potential target could be individuals who engage in sports regularly or follow balanced diets, who search for varied protein sources based on their origin [22].

The main components of insects are protein and fat, followed by fiber and carbohydrates. In particular, species from the order Orthoptera (grasshoppers, crickets, locusts) are rich in proteins and represent a valuable alternative protein source [24,25,26]. The nutrient quality of insect protein is promising in comparison to casein and soy, but varies and can be improved by the removal of the chitin [27]. Furthermore, most edible insects sufficiently provide the required essential amino acids [26,28]. Fat represents the second largest portion of the nutrient composition of edible insects, ranging from 13.41% for Orthoptera (grasshoppers, crickets, locusts) to 33.40% for Coleoptera (beetles, grubs). The fatty acids of insects are generally comparable to those of poultry and fish in their degree of saturation, but contain more polyunsaturated fatty acids (PUFA) [25,26]. The ratio of ω-6 and ω-3 fatty acids of edible insects is mostly 5:1 to 5.7:1 [24,29], reducing proinflammatory profile, which contributes to the prevalence of atherosclerosis, obesity, and diabetes [30]. Carbohydrates are mostly formed from chitin. It is still unclear if chitin can be digested by humans [27] and its content depend on the species and the developmental stage [25]. Edible insects have the potential to provide with specific micronutrients such as copper, iron, magnesium, manganese, phosphorous, selenium, and zinc; 100 g of edible insects generally lack enough calcium and potassium and they can be utilized in low-sodium diets. In addition to minerals, edible insects can be rich in vitamins, but species have to be specifically selected for the provision of desired vitamins [25,26].

Notably, different kind of exercise produce acute changes on metabolism of several nutrients. Dietary requirements for athletes are widely studied [1], as well as nutritional strategies that combine different forms of supplements to maximize the bioavailability of nutrients within the periodized training program [31]. The nutritional profile of some species of insects could cover the nutritional needs of athletes, representing an alternative daily diet to traditional animal-based food. Energy-protein insect bars, properly balanced and enriched with carbohydrates, could represent an alternative to those already widely used on the market as supplements for sport.

As a result, the opportunity promoted in this study could be a new challenge in nutritional supplements for sportsmen, including professional athletes [22,32,33].

However, a consumer’s acceptance of insects as food is crucial in order to include this novel food in the diet. The most common barriers in explaining the aversion of Italian consumers towards eating insects are neophobia and disgust [20,23,34,35]. Although disgust is a universal emotion, it is important to note that the factors eliciting disgust can be different across individuals and cultures [36,37,38]. There is reason to believe that insects presented in different meal formats such as processed insect-based foods (e.g., snacks) might elicit more positive associations and taste expectations. Considering that negative taste expectation is a strong barrier to include a novel food [23], invisible insects as ingredients might reduce neophobic reactions and thus increase consumers’ willingness to try [23,34,39]. Moreover, several studies with Italian consumers [20,40] also showed that also curiosity about the sensory attributes and a focus on environmental benefits might be motivating factors to promote entomophagy.

To the best of the authors’ knowledge, no studies have focused on psychological and demographic predictors of sportsmen influencing the willingness to accept insect-based food.

In line with the growing interest in insects, consumption as food, and athletes’ attitude for consuming sports dietary supplements, the present study introduces a protein insect-bar to investigate dispositional traits and emotional factors in accepting insect-edible food as dietary supplements among professional athletes.

In line with previous studies [9,41,42,43,44], several factors have been investigated: dietary supplements consumption, nutritional knowledge, food neophobia, and individual factors influencing the willingness to consume insect-based protein bars as dietary supplements. Finally, we evaluated how a brief informative text on the environmental and nutritional benefits of using insects as food [34,45] impacts the acceptance of eating insect food, i.e., protein bars enriched with cricket-flour. 

## 2. Materials and Methods

### 2.1. Partecipants

Sixty-one professional athletes (27 females) aged 19 to 39 years (M = 27.8; SD = ±5.03) were recruited on a voluntary basis from the Italian Air Force Sports Centre in Rome. The average educational level was mainly high school (65.6%). The target population is made by professional athletes selected by the Italian National Sports Federations and most of them were currently competing at the international level (91.8%). We recruited mainly athletes from track and field (31.1%), fencing (23.0%), and beach-volleyball (18%); athletes from archery, sailing, skeet shooting, tennis table and artistic gymnastic were also recruited but in smaller numbers. The only inclusion criterion was that they had to be consumers of nutritional supplements. There were no age or gender restrictions. The exclusion criteria were (1) being vegetarians, (2) being in any concomitant nutritional counselling programs, (3) having any disease or health condition that required specialized dietary planning. Participants who had retired from a sport or had not participated in a competitive game or competition in the last year were excluded. Each participant was identified with an ID number to guarantee his/her anonymity.

### 2.2. Measures

A self-administered questionnaire was used with different sections:

(a) Information on socio-demographic characteristics, sports characteristics, knowledge and motivational aspects on nutritional supplement consumption, and dietary habits mainly related to animal protein food.
(b) The personal knowledge about general and sport nutrition (general nutrition knowledge—GNK and sport nutrition knowledge-SNK) by an adapted version of the Nutrition for Sport Knowledge Questionnaire (A-NSKQ) [46,47]. Total scores were assessed using one point for each correct answer, no negative points, and coding “unsure” answers as incorrect. The total score was out of 37. All domains were weighted equally during scoring, and percentages were determined. The following cut-off points were used poor knowledge (0–49%), average knowledge (50–65%), good knowledge (66–75%), and excellent knowledge (over 75%).
(c) Food neophobia was evaluated by the Food Neophobia Scale (FNS) [48]. It consists of 10 statements, five neophilic and five neophobic statements about food or situations related to food consumption, rated on a 7-point scale ranging from 1 = strongly disagree to 7 = strongly agree. After reverse coding the responses for the neophilic statements, a total FNS score ranging from 10 to 70 was then calculated by summing the ratings for each item; the higher the FNS score, the higher the food-neophobia level. According to recent studies, the consumers were categorized as having a low, medium or high level of food neophobia, and sustainable behavior. The frequency distribution of the FNS scores was calculated and the subjects were divided into the following three groups: “low neophobia” (subjects in the lowest quartile, FNS scores ≤ 23), “medium neophobia” (subjects in the second and third quartile, FNS scores ≥ 24 and ≤41) and “high neophobia” (subjects in the highest quartile, FNS scores ≥ 42) [49].
(d) Athletes’ willingness to taste an insect-based protein bar was evaluated using a 7-point Likert scale (1 = strongly disagree, 7 = strongly agree). The population was split between groups: willing (from 5 to 7 points), uncertain (point 4) and unwilling (from 1 to 3 points).
(e) A brief informative text on the environmental and nutritional benefits of edible insects was provided to the participants (Table 1). After reading the text, participants were asked to assess their degree of agreement/disagreement in tasting the product.
(f) After the athletes expressed their willingness to taste the insects, two separate groups were identified (taster/no taster) and the factors which influenced their choice, i.e., curiosity about the texture, palatability, and alternative protein source for the tasters, and disgust, suitability for society, personal diet, poor hygiene and fear of unpleasant taste for the no tasters, were investigated using a 7-point Likert scale.
(g) Finally, athlete-endorsements in the food market were also explored using the following question: “from what it has been described above, how much would you be willing to promote this novel food product?” A 7-point Likert scale was used (1 = strongly disagree, 7 = strongly agree). The population was split between groups: willing to endorse (from 5 to 7 points), uncertain (point 4) and unwilling to endorse (from 1 to 3 points).

Informed consent was obtained from all human research participants. The study was conducted in accordance with the Helsinki declaration and the ethical rules of the Italian Psychological Association.

### 2.3. Statistical Analysis

The Statistical Package for Social Science (SPSS 25) was used to analyze the data for all variables. Descriptive statistics were run to summarize the data collected and the results were displayed in frequencies and percentages. Friedman’s and Cochran’s Q test were run to determine if response for each category differed significantly. The internal consistency of the multi-scales was measured with Cronbach’s α coefficient. Association between groups were calculated by Pearson’s correlation or Spearman’ correlation (if normal data distribution was not obtained or for ordinal variables). Differences between groups were calculated by independent-sample *t*-test, one-way-ANOVA, and a Mann–Whitney U test (if normal data distribution was not obtained) and one-way Welch ANOVA if there was heterogeneity of variances, assessed by Levene’s test. The influence of various socio-demographics variables on food neophobia and A-NSKQ was examined using multiple linear regression analysis. A two-way repeated measures ANOVA was run to determine the effect of information treatment on the willingness to taste a cricket bar over time. The Shapiro-Wilk test (*p* < 0.05) was conducted on the difference scores to ensure normality for all variables with significant main effects. For normally distributed variables with significant main effects, post hoc dependent t-tests were conducted and effect sizes (Cohen’s d) were calculated. Effect sizes were interpreted as small (0.20), medium (0.50), and large (0.80). For any variables that was not be normally distributed, the Wilcoxon signed-rank test and Glass’s delta (effect size) were utilized for post hoc contrasts. In all tests, *p* < 0.05 was considered statistically significant.

## 3. Results

### 3.1. Dietary Habits and Nutrition Supplements

All 61 athletes consumed nutritional supplements, such as protein-amino acid supplements (68.6%), multivitamins (64%), minerals (44.3%), sport bars (42.6%) and fish oils (39.3%), χ^2^ (3) = 12.7, *p* < 0.001. The prevalence of supplement consumption among professional athletes in the current study is similar compared to previous reports [2,9,10]. The qualified personnel (nutritionist/dietician/physician/pharmacy) were the most common source of information regarding nutritional supplements (88.5%) among athletes, χ^2^ (4) = 52.61, *p* < 0.001. Most of the athletes used supplements to improve their recovery (72.1%), improve performance (49.2%), and health (45.9%) and to prevent deficiencies (36.1%) (Supplementary Table S1).

### 3.2. Neophobic Scale

Participants showed a mean value in FNS of 33.6 (SD 11.4). Subjects with “low neophobia” comprised 19.7% of the sample, “medium neophobia” comprised 55.7%, and “high neophobia” comprised 24.6%. Internal consistency was acceptable for food neophobia multi-scales (Cronbach’s α = 0.85). Moderate correlation was found between age and FNS, r (59) = 0.3, *p* < 0.05. A linear regression established that age could statistically significantly predict FNS, F (1, 25) = 4.83, *p* < 0.05 and age accounted for 16% of the explained variability in FNS. The regression equation was: FNS = 8.8 + 0.99× (age).

### 3.3. Abridged–Nutritional Sport Knowledge Questionnaire (A-NSKQ)

Professional athletes in this study showed a poor nutritional sport knowledge (47.8%). Internal consistency was acceptable for A-NSKQ multi-scales (Cronbach’s α = 0.82). There was a large variability amongst participants and between SNK and GNK and several misconceptions were evident in A-NSKQ, especially with regards to hydration, micronutrients and proteins (Supplementary Table S2).

Weak correlation was found between age and A-NSKQ, r (59) = 0.29, *p* < 0.05. An ANCOVA was run to determine the effect of level of education and ANSKQ after controlling for age. After adjustment for age, there is not a statistically significant difference in ANSKQ score, F(1, 39) = 2.34, *p* = 0.134, partial η^2^ = 0.057.

### 3.4. Willingness to Taste the Cricket Flour Enriched Bar

Participants were classified into three groups based on their willingness to taste the cricket flour enriched bar: willing (*n* = 26), uncertain (*n* = 7), unwilling (*n* = 28), with a total mean value of 3.9 (SD 2.1). There were statistically significant differences in food neophobia between the different groups, F(2, 58) = 7.045, *p* = 0.002. Regression analysis indicated that 29% of the variability on willingness of tasting before the information was significantly accounted for by FNS, F(1, 59) = 24.3, *p* < 0.001, adj. R^2^ = 0.28.

**Figure 1:**
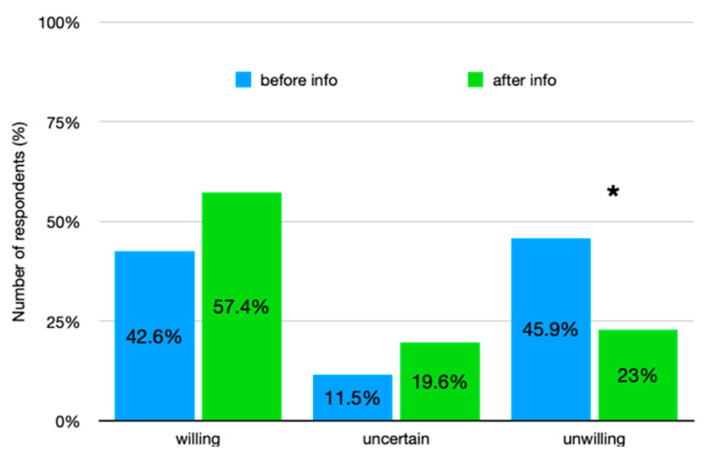
Willingness to taste an energy bar enriched with cricket flour. Difference in number of respondents (%) on the willingness to taste the cricket bar, before and after the information, among three different groups. Groups were defined using a 7-point Likert scale (1 = strongly disagree, 7 = strongly agree): willing (from 5 to 7), uncertain (point 4) and unwilling (from 1 to 3); number of respondents of the unwilling group decreased significantly * *p* < 0.001.

**Figure 2:**
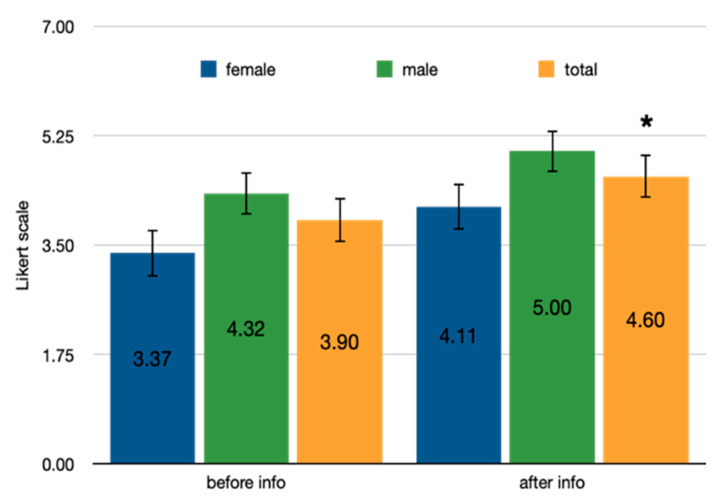
Willingness to taste an energy bar enriched with cricket flour, pre and post treatment. Difference in mean score in Likert scale, ranging from 1 = strongly disagree to 7 = strongly agree on the willingness to taste the cricket bar, before and after the information, among gender and the whole sample; willing to taste significantly increases in the reference sample * *p* < 0.001.

Following the information treatment, there was a statistically significant median increase in acceptance of the insect-bar (z = 4.16, *p* < 0.001) in number of respondents, in particular among the unwilling athletes, z = 3.88, *p* < 0.001 (Figure 1). A Wilcoxon signed-rank test determined that there was a statistically significant median increase in willingness to taste among the participants, rated on a 7-point scale (0.71), z = 4.16, *p* < 0.001 (Figure 2).

**Figure 3:**
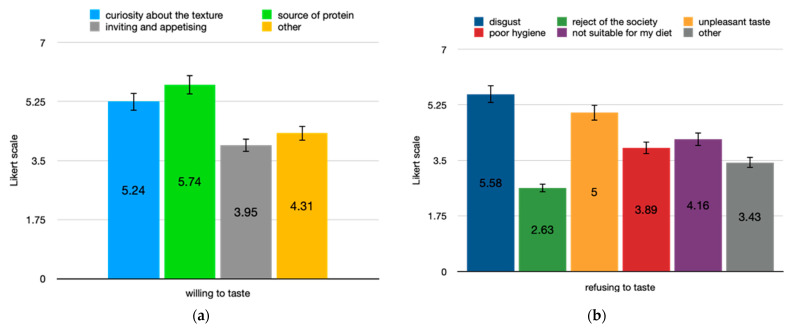
Reasons to taste or refuse cricket bar among the athletes. Difference in mean score in Likert scale, ranging from 1 = strongly disagree to 7 = strongly agree on the motivations (**a**) to taste or (**b**) refuse the cricket bar.

Finally, two groups were identified (taster/refusing): 43 athletes (70.5%) were willing to taste the cricket-bar, 15 females and 28 males (.3, *p* = 0.011) and the remaining 18 were not willing. The reasons to taste were investigated and the response for each category differed significantly, χ^2^ (3) = 18.1, *p* < 0.001. A regression analysis indicated that 31% of the variability in the willingness to taste the product was significantly accounted for by “alternative research of a protein source”, F(1, 11) = 6.35, *p* < 0.05, adj. R^2^ = 0.3. Afterwards, factors influencing “no taster” group were investigated, the response for each category differed significantly, χ^2^ (4) = 41, *p* < 0.001. Regression analysis indicated that 38% of the variability in the rejection to taste the product was significantly accounted for by “disgust”, F(1, 8) = 6.5, *p* < 0.05, adj. R^2^ = 0.38 (Figure 3).

### 3.5. Athletes Endorsement in Cricket-Bar Marketing

**Figure 4:**
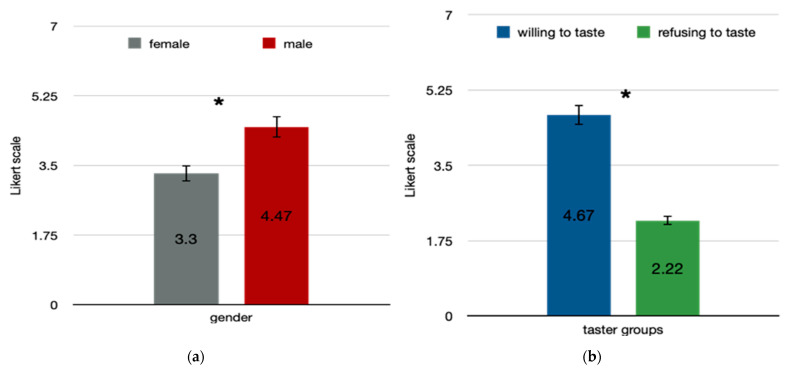
Willingness to endorse an energy bar enriched with cricket flour. Difference in mean score in Likert scale, ranging from 1 = strongly disagree to 7 = strongly agree on the willingness to endorse the cricket bar among (**a**) gender and (**b**) taster groups; males and willing to taste group were significantly prone to endorse the product * *p* < 0.01.

In this study, 37.7% were willing to endorse the cricket bar. The taster group was more willing to endorse (4.67 ± 1.34) the cricket bar than no-taster group (2.22 ± 1.11), a statistically significant difference of 2.45 (95% CI, −3.17 to −1.73), t(59) = −6.828, *p* < 0.001. Moderate negative correlation was found between athletes-endorsement and FNS score, r (59) = 0.3, *p* < 0.05. Regression analysis indicated that 10% of the variability in the willingness to sponsor the product was significantly accounted for by NS, F(1, 59) = 6.78, *p* < 0.05, adj. R^2^ = 0.9. Males were more willing to endorse (4.47 ± 1.69) the cricket bar than females (3.3 ± 1.49), a statistically significant difference of 1.17 (95% CI, −2.01 to −0.35), t(59) = −2.365, *p* = 0.006 (Figure 4).

## 4. Discussion

This study investigated the potential motivations to accept eating an energy protein bar enriched with cricket flour among athletes, considering that motivation is a strong determinant in the type of supplement used [50,51]. In line with previous studies, the main motivations among athletes for the use of supplements in general included improving performance and recovery from exercise and preventing deficiencies. Protein, vitamin, minerals, and fish oils supplements were the most frequently used by athletes and recommended by acquired from qualified personnel (nutritionists/dieticians and physicians). This is important if we consider the vulnerability of the participants and their limited knowledge in sports nutrition [2]. Moreover, the high number of products available, often without a guarantee of accurate information, may increase the risk of using illegal substances, i.e., doping [6,7,41,52]. Our results support what has already been underlined in previous studies, i.e., the promotion of additional nutrition training as part of continued professional development of coaches and trainers would ensure better preparation to address nutritional concerns for athletes [53,54].

When asked about food neophobia, only 24.6% of athletes showed high neophobia and it could reflect exposure to different dishes during numerous contacts with foreign cultures during international events they attend [55]. In line with past studies [20,23,38], age was confirmed to be positively associated with food neophobia. No significant difference was found in food neophobia between gender; however, women seemed to be less likely to accept the energy bar with insect flour, probably due to their higher insect aversions than men [18,56,57]. It is likely that the high level of competition tends to make the difference between genders in supplement consumption less significant. Nevertheless, female athletes in this study report the same adversity towards insects as the common population.

Our results shown that food neophobia negatively contributes to the intention to taste and endorse the insect bar, and disgust was the main factor that determined the rejection of tasting. According to our data, food neophobia, even if it corresponds to the refusal to introduce insects into the diet, does not represent a characteristic of this type of population. Rather, it would be more relevant to investigate the disgust factor in athletes. After all, La Barbera et al. [35] demonstrated that the explanatory power of disgust towards the intention to consume insects was even higher than the explanatory power of food neophobic tendencies. Professional athletes have an important opportunity to promote the public’s health, particularly for youth, by refusing endorsement contracts that involve promotion of energy-dense, nutrient-poor foods and beverages. Environmental sustainability and health promotion represents the main benefits of insects as food. However, only 37.7% of athletes willing to endorse the product, mainly males. Nevertheless, athletes who expressed the willingness to taste the product were more likely to endorse it. Reducing food neophobia could increase their willingness to endorse cricket bars as a new form of supplement. We believe that this sector should invest in sensory and gastronomic features, as well as advertising messages to reduce food neophobia and disgust about insects as food [23,39,45].

In line with previous consumer studies [37,40], our data indicated that the main factors for trying the insect-based product are the high protein content and the curiosity about the texture. Reinforcing the association between the relevant protein intake, i.e., nutrition profile, in a familiar product (energy bar) could increase the acceptability of insects as supplements in this target population.

Athletes have always shown a greater focus on high protein foods and strategies aimed at the consumption of protein-enriched foods or supplements. From a managerial point of view, planning proper marketing campaigns is supposed to reinforce the association between high protein content or alternative protein source and insect-based foods in the minds of this type of population.

As for the general population, taste has a strong influence on food choice, however, it may become less critical prior to an important game or event when foods that benefit performance are preferred, particularly in a highly competitive sport. The limited number of studies with athletes has reported performance or competition are one of the most important influences on food choice [8].

Our results shown a significant variation in the willingness to taste before and after the information treatment, in line with previous studies [45,49,58,59]. The athlete acceptability of insect-based foods was systematically increased after receiving information about the potential environmental and nutritional benefits of including insects in the diet.

Consequently, although recent technological developments in assuring food security for edible insects, e.g., automation and reduction in microbial contamination by personnel [16,19], the future marketing of these products in professional sport must also ensure that the products do not lead to any anti-doping rule violation.

This study has several limitations. First, it is a small sample for a quantitative study, and therefore, we are aware that generalization of the findings is difficult and should be done with caution. However, this is due to the specific target under investigation (professional athletes) which is not allowing to recruit a higher number of respondents. Thus, an extension of the dataset, including the widest variation possible for the sample, may increase the quality of future studies. Moreover, the comparison in nutritional knowledge with other studies was difficult, due to the heterogeneity of the measures used and reduced number of articles. The questionnaire only measured self-reported willingness to try insects and did not observe actual behavior to eat insects. In fact, in our study, it was not possible to carry out a “bug experience” that they might be categorized as “hidden substances” considered as high risk of doping in this specific category of participants. It is important to identify the species of insect best suited to cover the nutritional needs of athletes, reducing the risk of doping and optimizing training sessions as well as recovery and metabolic adaptation.

Future studies should consider using real products (e.g., bars with cricket flour) to measure actual consumption of insect food [18] and also include the Entomophagy Attitude Questionnaire Scale [60] instead of the FNS scale, to better investigate the individual aversion to eating insects.

## 5. Conclusions

This study represents a novelty in the evaluation of the acceptance of edible insects, for the choice of professional athletes as a sample and for the use of informative treatments in this type of subject. Investigating endorsements for edible insect among athletes could represent a new strategy to promote environmental and nutritional education in ordinary people. Insect protein could represent a new option to deliver nutrients to individuals who practice exercise and sport activity for work. Moreover, usually society holds a positive image towards professional athletes, and thus, these individuals could represent a target to promote the integration of insect-based products into sports nutrition.

Providing information about the environmental and nutritious benefits of edible insects will reduce food neophobia and disgust and favor acceptance of insects as food among individuals.

## Figures and Tables

**Table 1:** The information text provided to the sample.

In recent years, several European countries have begun to sell edible insects in supermarkets. Energy or protein bars are produced in a certified way, using insect’s flour as supplements and their use is already widespread among athletes all over the world. From a nutritional point of view, insects are rich in proteins, minerals and vitamins, have a low-fat content and a reduced caloric intake, all elements that identify them as complete and healthy foods. Furthermore, insects farming has a lower environmental impact (e.g., few resources needed to raise them and reduced emission of carbon dioxide) compared to domestic animals.

## Data Availability

The data presented in this study are available upon reasonable request from the corresponding author.

## Supplementary Materials

The following are available online at https://www.mdpi.com/article/10.3390/foods10051117/s1, Table S1: responses (percent correct) of individual items in the use of nutritional supplements; Table S2: Responses (percent correct) of individual items in the A-NSKQ.
