# Supplementary material for: The New Challenge of Sports Nutrition: Accepting Insect Food as Dietary Supplements in Professional Athletes
Source: Foods. 2021 May 18;10(5):1117. doi: 10.3390/foods10051117 (PMC8157859; doi:10.3390/foods10051117)
Supplement: Supplementary file 1 [file foods-10-01117-s001.zip › foods-1194162-supplementary.pdf]

Supplementary Materials

Table S1. Responses (percent correct) of individual items in the use of nutritional supplements

Distribution of responses to reasons, source of information, type of supplements and perceptions about nutritional (N = 61)	
**What is the main reason of using dietary supplements? n (%)**	
Improve performance	30.0 (49.2)
Prevent deficiencies	22.0 (36.1)
Improve health	28.0 (45.9)
Improve recovery	44.0 (72.1)
Gain weight & muscle	11.0 (18.0)
Recover from an injury or illness	7.0 (11.5)
Supplement diet	8.0 (13.1)
**How do you obtain information about these products? n (%)**	
Coach/instructors	24.0 (39.3)
Friends/Teammates	9.0 (14.8)
Retail store/pharmacy	4.0 (6.6)
Nutritionist/dietician	40.0 (65.6)
Physician	21.0 (34.4)
Magazine/online	5.0 (8.2)
**Which of nutritional supplements have you used most frequently? n (%)**	
Multivitamins	39.0 (64.0)
Fish oils: omega3,6	24.0 (39.3)
Minerals	27.0 (44.3)
Protein-amino acid supplements	42.0 (68.9)
Herbals	5.0 (8.2)
Creatine	15.0 (24.6)
Sport bar	26.0 (42.6)
Carbohydrates supplements or sugar	20.0 (32.8)
**What is the perceptions about supplements? n (%)**	
Make me healthier	28.0 (45.9)
Improve my endurance	20.0 (32.8)
Improve my speed	2.0 (3.3)
More energy, increasing the amount of training I can do	38.0 (62.3)
Are safe to use	11.0 (18.0)
May contain doping agents	2.0 (3.3)
Improve my concentration	7.0 (11.5)
Help me train and compete	26.0 (42.6)
Increase my ability to cope with pain	1.0 (1.6)

Table S2. Responses (percent correct) of individual items in the A-NSKQ

**General Nutrition Knowledge**	Correct %	Incorrect %	Uncertain %
Protein eaten in excess of bodily needs can lead to fat gain	44.1	44.1	11.9
Do you think these foods are high or low in carbohydrate? A Banana	67.8	22.0	10.2
Do you think these foods are high or low in carbohydrate? 1⁄2 cup cooked quinoa	45.8	47.5	6.8
Do you think these foods are high or low in fat? 1 TBS honey	67.8	15.3	16.9
Fat is required by the body to make cell membranes and molecules involved in immune function	42.4	50.8	6.8
Do you think these foods are high or low in fat? 1/2 Cup Cottage cheese	67.8	23.7	8.5
Do you think these foods are high or low in fat? 1 TBS Polyunsaturated margarine	89.8	8.5	1.7
Protein absorption in a single sitting is limited	72.9	25.4	1.7
Do you think these foods are high or low in protein? 30g (1 ounce) Yellow Cheese	57.6	39.0	3.4
Do you think these foods are high or low in protein? 1 Cup Baked Beans	84.7	11.9	3.4
Do you think these foods are high or low in protein? 1/2 Cup Cooked Quinoa	54.2	40.7	5.1
Eggs contain all the essential amino acids needed by the body	52.5	22.0	25.4
Thiamine (Vitamin B1) is required for efficient delivery of oxygen to muscles	8.5	32.2	59.3
Vitamins provide the body with energy (kilojoules/calories)	55.9	35.6	8.5
When consumed as part of the diet, pure alcohol (ethanol) contains calories/kilojoules and, therefore, can lead to weight gain	78.0	16.9	5.1
Drinking large amounts of alcohol can reduce recovery from injury	78.0	16.9	5.1
"Binge drinking" (also referred to as heavy episodic drinking) is generally defined as	44.1	52.5	3.4
**Sport Nutrition Knowledge**			

Increasing protein in the diet is the main dietary change needed when only muscle gain is desired	11.9	86.4	1.7
Which do you think is the best lunch option for an athlete trying to gain weight (muscle)? Assume they are training in the morning and have already had breakfast and a mid-morning snack:	40.7	57.6	1.7
When exercising at low intensities, fat provides almost all the substrate needed to cover energy costs	47.5	39.0	13.6
Vegetarian athletes can meet their protein requirements without the use of protein	45.8	45.8	8.5
The protein needs of a 100 kg (220 lb) well trained resistance athlete are closest to:	30.5	45.8	23.7
Athletes have increased magnesium needs due to losses in sweat	10.2	79.7	10.2
The optimal calcium intake for athletes aged 15 to 24 years is 500 mg	10.2	22.0	67.8
A physically fit person eating a nutritionally adequate diet can improve their performance by eating more vitamins and minerals	39.0	57.6	3.4
Vitamin C should be routinely supplemented by athletes	27.1	61.0	11.9
Athletes should drink water during activity in order to:	20.3	71.2	8.5
Regarding fluid intake during physical activity, current recommendations encourage athletes to:	16.9	72.9	10.2
Before competition, athletes should aim to consume foods that are high in:	45.8	52.5	1.7
In events last 60 - 90 minutes, 30- 60 g (1.0 - 2.0 ounces) of carbohydrates should be consumed per hour	55.9	15.3	28.8
Consuming carbohydrate during exercise will assist in maintaining blood glucose levels	79.7	3.4	16.9
Which of the following best meets the recommendations for a snack consumed during high-intensity exercise lasting around 90 minutes?	47.5	49.2	3.4
How much protein do you think experts recommend athletes should have after completing a resistance exercise session?	37.3	40.7	22.0
Supplement labels may contain false or misleading information	52.5	30.5	16.9

The purity and safety of all supplements are tested before sale	44.1	42.4	13.6
In relation to improving sporting performance, which of the following supplements do you think has NOT been supported by a strong body of scientific evidence?	5.1	39.0	55.9
Which of the following supplements do you think is banned by the WORLD ANTI-DOPING AGENCY (WADA)?	91.5	8.5	0.0
